# Numerical Simulation of the Hot Rolling Process of Steel Beams

**DOI:** 10.3390/ma14227038

**Published:** 2021-11-20

**Authors:** Alejandro Pérez-Alvarado, Sixtos Antonio Arreola-Villa, Ismael Calderón-Ramos, Rumualdo Servín Castañeda, Luis Alberto Mendoza de la Rosa, Kinnor Chattopadhyay, Rodolfo Morales

**Affiliations:** 1 Mechanical Engineering Department, Universidad Autónoma de Coahuila FIME–U.N., Barranquilla S/N, Col. Guadalupe, Monclova 25750, CP, Mexico; alejandro.perez@uadec.edu.mx (A.P.-A.); i.calderon@uadec.edu.mx (I.C.-R.); rumualdo.servin@uadec.edu.mx (R.S.C.); luismendoza@uadec.edu.mx (L.A.M.d.l.R.); 2 Department of Material Science and Engineering, University of Toronto, 184 College Street, Toronto, ON M5S 3E4, Canada; kinnor.chattopadhyay@utoronto.ca; 3 Department of Metallurgy and Materials Engineering, Instituto Politécnico Nacional–ESIQIE, Ed. 7, Col. Zacatenco, Mexico City 07738, CP, Mexico; rmorales@ipn.mx

**Keywords:** hot rolling process, numerical simulation, thermo-mechanical deformation

## Abstract

The complete rolling schedule (25 passes) of steel beams in a mill was simulated to predict the final beam length, geometry of the cross-section, effective stress, effective plastic strain and rolling power for two cases; the first case corresponds to the hot rolling process assuming a constant temperature of 1200 ${}^{\circ }C$. The simulation of the second case considered the real beam temperature at each pass to compare the results with in-plant measurements and validate the numerical model. Then, the results of both cases were compared to determine the critical passes of the process with high peaks of required power, coinciding with the reports at the mill. These critical passes share the same conditions, high percentage of reduction in cross-sectional area and low beam temperature. Additionally, a potential reduction of passes in the process was proposed identifying passes with low required power, minimal reduction in area of cross-section and essentially unchanged geometry. Therefore, it is reasonable to state that using the present research methodology, it is possible to have a better control of the process allowing innovation in the production of profiles with more complex geometries and new materials.

## 1. Introduction

Steel is the most important metal used for structural and mechanical purposes because it combines high strength, both in tension and compression, with great rigidity (high modulus of elasticity) and easy manufacture, with a relatively low price. In addition, steel is a ductile material by nature, which has a stable behavior under load inversions, presenting a very favorable resistance-weight ratio for structural applications. On the other hand, the mechanical properties of steel beams for structural purposes are directly influenced by the hot rolling parameters such as strain, strain rate, groove design, rolling sequence and the most important, the workpiece temperature during the entire process. In hot rolling, the desired shape is obtained by pulling the metallic material by the effect of friction force between two rolls rotating in opposite directions. These rolls have grooves with the dimensions designed to obtain the desired shape [1]. To achieve a successful beam fabrication through the hot rolling process, it is necessary to design a correct rolling sequence. However, complicated mechanics involved in shape rolling have made the design process an art of experience. Therefore, it is very common to rely on expert’s decisions in process design, especially for complex shapes. However, for simple shapes, empirical rules and formulas stored in a personal computer can considerably reduce such an experience-oriented design procedure [2]. Today, due to global competition, companies need to produce with higher quality and lower costs. Thanks to the simulation tool, it is possible to quickly and inexpensively determine if a process is correct. In this regard, it allows conducting experiments without wasting excessive time and resources. Furthermore, simulations could aid to improve quality and process times of hot rolling. In addition, dangerous, difficult to access/control or new designed systems can be simulated, thus, avoiding to risk lives in the real process.

Investigations have been carried out to understand the fundamentals of the hot-rolling process. Mori et al. [3] simulated steady and unsteady strip rolling of aluminium using the finite element method (FEM). They found good agreement when comparing the front and back-end profiles obtained from the simulations with those of experimental data. During hot rolling high reductions in the initial passes are beneficial to promote void closure, grain refinement, reduce the number of passes, and minimize crack formation and fish tail [4,5]. Lee et al. [6] studied the mean effective strain in a four-pass rolling sequence. Their experimental results validated an analytical model for rod and bar experiencing similar strain in hot rolling. In a later investigation, Lee et al. [7] developed a mathematical model to compute thermo-mechanical parameters (temperature, strain and strain rate) as well as austenite grain size for the same four-pass rolling sequence finding good agreement between experiments and simulations. Mróz et al. [8] used FEM to simulate one pass of hot rolling of bars considering a non-uniform temperature distribution along the bar (to represent irregular heating of the material previous to the process). The results showed variations in the rolling torque attributed to the non-uniform temperature and in-plant measurements showed a similar behavior. Shahani et al. [9] employed FEM to simulate one pass of hot rolling of AA5083 aluminum alloy. In their analysis, the main variables of the process were expressed in parametric form and the results of temperature distribution, stress and strain were in agreement with previous published data. Licheng et al. [10] simulated one pass of hot rolling of steel strips using FEM to obtain the stress and strain fields for different reduction ratios and work roll radii. The results showed higher contact stresses and strain in all the strip when increasing roll radius and reduction ratio, respectively. Nalawade et al. [11] simulated eight intermediate passes of hot rolling of 38MnVS6 steel using a FEM. The results of surface temperature, load, cross-section and macrostructure were in good agreement with experimental measurements. In another work, Nalawade et al. [4] employed FEM to simulate the initial four passes of hot rolling of steel. They investigated variations in the geometry of the groove and the results showed that the largest groove depth and the lowest taper collar angle investigated required lower load and reduced fish tail formation. Braga et al. [12] analysed recrystallization on Nb-stabilized AISI 430 ferritic stainless steel by applying hot torsion to simulate finishing rolling. The experiments were carried out at different temperatures and the results suggest that high temperature at the first passes would be beneficial to allow recrystallization while lower temperatures at the last passes are desirable for work hardening. Rout et al. [13] studied the hot cross rolling of 304 stainless steel plates using FEM and experiments. They analized the flow of material for two passes and compared the profiles of the edges of the simulations and experiments. They found good agreement between the measured and simulated profiles, however, deviations at the corners were observed. Hanoglu et al. [14] simulated the rolling of non-symmetric profiles applying a slicing model. The profiles obtained in the simulation were in good agreement with the profiles of the actual mill. The roll’s grooves and pass sequence of the hot rolling process are designed considering the characteristics of the material, dimensions of the final product and mill capacity. Analytical and empirical methods have been generally used, however, the current advances in the computational capabilities and simulation methods allow to improve the design of the hot rolling process in a faster, safer, and relatively inexpensive way. Many publications are available in the field of deformation of metals as well as rolling simulations, however, there are not enough studies of its complete rolling sequence using the real billet temperature during operation time. This research aims to contribute to the development of roll pass design and the understanding of the effect of the billet temperature, comparing experimental data from plant and the complete rolling sequence simulations using the FEM, to achieve a methodology to minimize time and costs of trial production, process optimization and new product design.

## 2. Materials and Methods

In the present study FEM based forming simulation software was used to analyze the metal behavior in the hot rolling of steel I-shaped rectangular skate (IPR) beams. The hot-rolling of the product identified as $\mathrm{IPR}18\times 7\frac{1}{2}$ comprises the use of two mills (U1 and U2). In mill U1 the majority of the passes are performed and the desired I-shaped profile is obtained, while mill U2 is used for finishes after mill U1. In this study, the rolling sequence of mill U1 will be investigated because it is the critical part of the process in which the significant changes in geometry are performed. In the simulations, the rolls are considered as rigid bodies while the workpiece is deformable using its mechanical properties to feed the model. The elastic effect is considered negligible for the billet. The governing equations of the metal deformation are based on the three-dimensional isotropic viscoplastic model of the Norton Hoff law [15] (Equation (1)).

(1) $$S=2K{\left(\sqrt{3{\dot{\varepsilon }}_{eq}}\right)}^{m-1}{\dot{\varepsilon }}_{vp}$$

where *S* is the Deviatoric stress tensor, ${\dot{\varepsilon }}_{vp}$ is the viscoplastic strain rate, ${\dot{\varepsilon }}_{eq}$ is the equivalent strain rate, *K* and *m* are the material parameters.

The strain rate is calculated as the sum of the elastic and viscoelastic components as shown in Equation (2).

(2) $${\dot{\varepsilon }}_{eq}={\dot{\varepsilon }}_{e}+{\dot{\varepsilon }}_{vp}$$

where ${\dot{\varepsilon }}_{e}$ is the elastic strain rate and ${\dot{\varepsilon }}_{vp}$ is the viscoplastic strain rate. In a Norton Hoff model, the elastic strain rate (${\dot{\varepsilon }}_{e}$) is assumed to be zero. This assumption is a good approximation at high temperature.

The viscoplastic strain rate is aproximated by a power law (Equation (3)).

(3) $${\dot{\varepsilon }}_{vp}=\frac{1}{K}{\left[\frac{\bar{\sigma }-R}{K}\right]}^{\left(\frac{1}{m}-1\right)}\overset{‘}{\sigma }$$

where $\bar{\sigma }$ is the equivalent stress, $\overset{‘}{\sigma }$ is the Deviatoric stress tensor, *m* is the strain rate sensitivity and *K* is the consistency constant. The flow stress at high temperature at given strain and strain rates is determined with the appropriate flow curves for the material in the FEM software. The plastic material properties are loaded from the database of the FEM software, these properties change with temperature and are assigned automatically for each pass.

The temporal evolution of the temperature during the hot rolling is calculated using the general heat Equation (Equation (4)) with internal heat generation.

(4) $$\rho c\frac{dT}{dt}=\nabla \cdot \left(k\nabla T\right)+{\dot{q}}_{v}$$

The boundary conditions were incorporated in the model with Equations (6) and (7). The heat transfer between the beam and rolls was evaluated by the following equation:

(5) $$\varphi =-k\nabla T=\alpha \left(T-T_{\infty }\right)$$

where α is the heat transfer coefficient. The heat transfer by radiation at the surfaces was calculated with the following equation:

(6) $$-k\frac{dT}{dt}=\varepsilon_{r}\sigma_{r}\left(T^{4}-T_{\infty }^{4}\right)$$

where $\sigma_{r}$ is the Stefan–Boltzmann constant, $\varepsilon_{r}$ is the emissivity, and $T_{\infty }$ is the temperature of the surroundings.

The friction shear stress at the interface between the beam and rolls was modeled by a viscoplastic Coulomb law (Equation (7)) as used by Duan et al. [16].

(7) $$\tau =\frac{-\alpha_{f}\left|\sigma_{n}\right|\Delta V}{{\left|\Delta V\right|}^{1-p}}$$

where $\alpha_{f}$ is the friction coefficient. The value of the friction coefficient is incorporated by using a model that considers the temperature and rolling velocity. It is the same model used in reference [17].

(8) $$\alpha_{f}=0.8\left(1.05-0.0005T-0.56v\right)$$

where *T* is the temperature and *v* is the rolling speed.

A 3D hexaedral element was employed to mesh the complicated geometry of the beam (especially when deformated). A mesh refinement analysis was performed for the first pass and the analysis showed that a 38 mm mesh element size was adequate. The model is able to regenerate the mesh to replace the excessively distorted elements during the simulation of the rolling process. The FEM mesh of the billet consisted of 6731 elements for the first pass and the final number of elements was 270,673 for the pass 25. The rolls were considered as rigid bodies for the simulations. In the present work, the rolling mill configuration and the billet were modeled using SolidWorks^®^ 2020 software and then exported to the FEM software. The input parameters used in the simulations are given in Table 1.

An initial beam blank of 812 × 203 ${\mathrm{mm}}^{2}$ cross-section with material properties of steel AISI E52100 has been considered. The chemical composition is 1% C, 97% Fe, 1.4% Cr, 0.35% Mn, 0.025% P, 0.20% Si, and 0.025% S. The physical properties at high temperature are incorporated in the simulation by Simufact^®^ from its database. This database has been used in other investigations with the same material, for example Shah et al. [18] and the references therein. The thermal properties change with temperature and the software automatically assign the appropriate value of the property with the temperature corresponding to each pass. The initial rolling temperature was 1200 ${}^{\circ }C$ (as measured in-plant) with the above common input parameters and boundary conditions.

**Figure 1:**
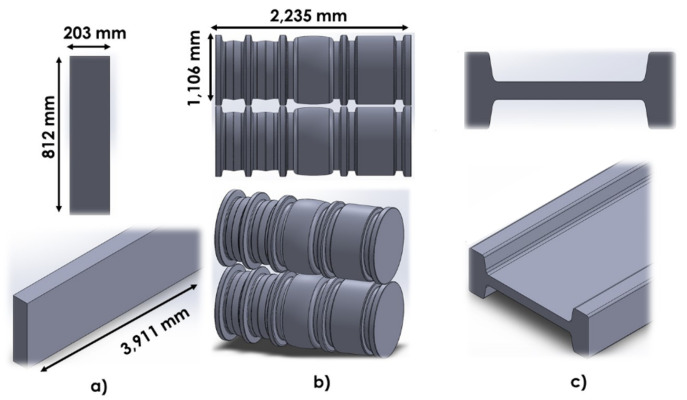
Geometry and dimensions: (**a**) beam blank; (**b**) rolls; (**c**) schematic of the IPR profile.

The elements used in the simulation consisted of a beam blank, top and bottom rolls. Rolls and beam blank geometries were created as a solid model in CAD program and then imported for analysis. Figure 1a,b show the rolls and the beam blank, respectively, meanwhile Figure 1c shows the schematic of the IPR profile obtained in the mill U1.

In this study, the hot-rolling of IPR profile was analyzed. Simufact^®^ Forming, a commercial finite element software, has been used for the simulations. The process starts with a draft profile (beam blank) with initial temperature of 1200 ${}^{\circ }C$ entering the gap between a pair of grooved rolls. With the help of a reversible mill and after 25 passes the IPR profile is manufactured. The simulation of each pass lasted from 1 to 15 h depending on the length of the beam in a Workstation Dell^®^ with a processor intel^®^ core i7 with 8 cores and 32 GB RAM. A total of 50 passes were simulated: one sequence (25 passes) of hot rolling considering constant temperature and another with variable temperature.

**Figure 2:**
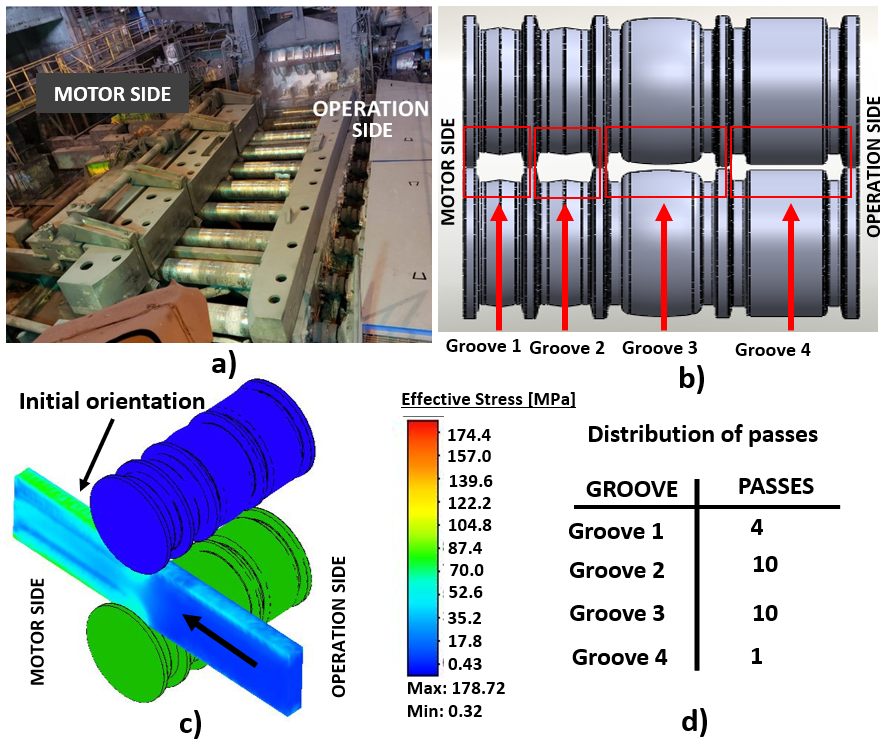
(**a**) Reversible hot mill; (**b**) location and geometry of each groove of the work roll; (**c**) initial orientation of the beam on the mill; (**d**) distribution of passes for each groove.

To recreate the real rolling process, the rolling schedule shown in Table 2 was used for the simulations. Figure 2 shows the actual mill, the geometry of the grooves, the initial orientation of the beam and the number of passes performed in each groove. Respecting the rolling schedule is of utmost importance for a successful lamination by achieving the correct flow of material when forming the profile.

**Figure 3:**
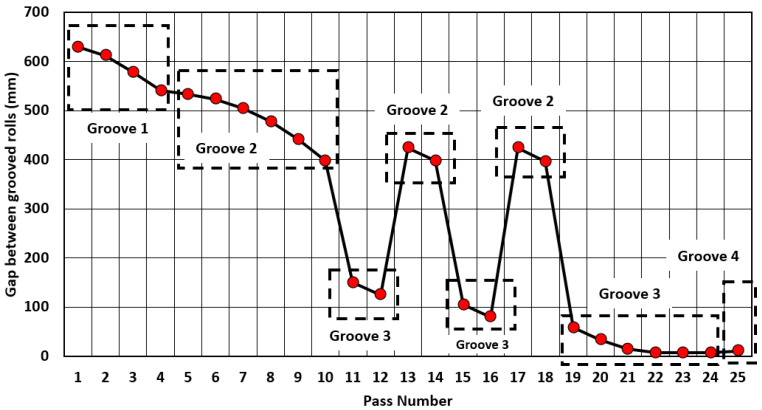
Gap between work rolls per pass and location of the beam through the rolling sequence.

Figure 3 shows the gap between the work rolls for each pass to understand the sequence and order of the groove used for the rolling passes. The geometric characteristics of the beam blank entering the U1 stand are: 812.8 mm wide, 203.2 mm high and 3911.60 mm long, equivalent to an initial cross-sectional area of 0.165 $m^{2}$, a volume of 0.646 $m^{3}$ and a mass of 5071.44 kg. These dimensions were used to predict the reduction percentage per pass (Equation (9)) and reduction ratio or final length per pass (Equation (10)) [11].

(9) $$\%R=\frac{(A_{0}-A_{f})\times 100}{A_{0}}$$

(10) $$L=\frac{V_{0}}{A_{f}}$$

where %R is the reduction percentage per pass, $A_{0}$ is the area before the pass, $A_{f}$ is the area after the pass, *L* is the final length after each pass and $V_{0}$ is the volume of the beam blank (this value is assumed constant). The values of the analytical %R and length of the beam per pass are shown in Table 3. To calculate these analytical quantities, it was assumed that the beam filled the entire groove in each pass and then the length was calculated by conservation of volume.

## 3. Results and Discussion

**Figure 4:**
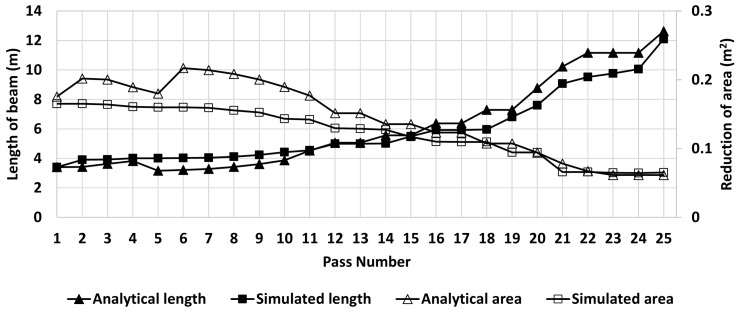
Comparison between analytical results and numerical results of the beam length at each pass.

The main objective of the present work is to validate the numerical model comparing the results of the simulations with those measured in-plant. The first step is to compare the calculated length and area of cross-section per pass with the predicted by the numerical model considering a constant temperature of the beam, Figure 4 shows the results obtained.

**Figure 5:**
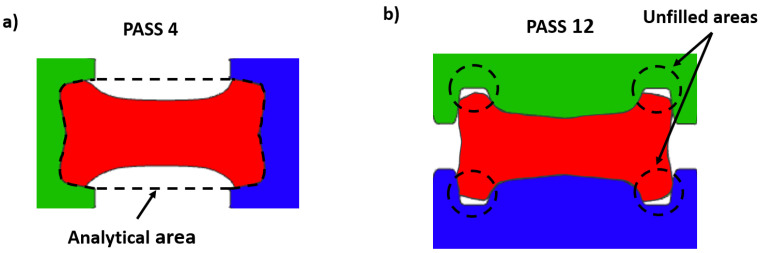
(**a**) Orientation of the beam in pass number 4; (**b**) unfilled areas at the grooved rolls during pass number 12.

Figure 4 shows that the analytical results have more abrupt changes in length, while the results of the numerical simulation show a smooth curve. This is because in the analytical calculations it was assumed that the beam filled the entire profile between the work rolls in each rolling pass; however, this does not happen neither in the simulation nor in the real process. During the first passes (in grooves 1 and 2), the beam undergoes slight deformations to roughly acquire the I-profile, and due to the orientation of the beam, it does not fill the groove. This situation is shown in Figure 5a for pass 4. Then, in grooves 3 and 4, the beam (rotated $90^{\circ }$ with respect to the orientation in grooves 1 and 2) is progressively filling the groove but there are small unfilled areas. Figure 5b shows unfilled areas for the simulation of pass 12. These observations demostrate that the simulation follows a physical mechanism and gradually fills the groove (i.e., not overestimating the rolling process). Therefore, it could be stated that the numerical model is accurate enough to represent and predict a real process.

The final ideal length of the beam (analytical result) is 12.65 m, while the final length predicted by the simulation is 12.10 m and the average final length reported in the real process is 12.35 m. The percentage of error between the ideal length and the length predicted by the simulation is 4.35%; while the percentage of error comparing the final length of the beam in the real process and the final length predicted by the simulation is 2.02%. This comparison validates the numerical model used in the present work.

The cross-sectional area in each pass is also shown in Figure 4. The difference of the simulation and analytical calculation is clearly observed in the first 10 passes. These passes are performed using grooves 1 and 2, and only an approximate I-shape is obtained when the rolls are in contact with a relatively small surface of the beam (see Figure 5a). Thus, the difference is the result of the overestimated analytical area. However, as the process continues in grooves 3 and 4, the difference is decreasing because the beam gradually fills the groove since there is more area of contact with the rolls (see Figure 5b). The curves overlap from pass number 18, indicating an adecuate design of the groove.

### Comparison between Hot Rolling Process at Constant Temperature and Real Temperature

**Figure 6:**
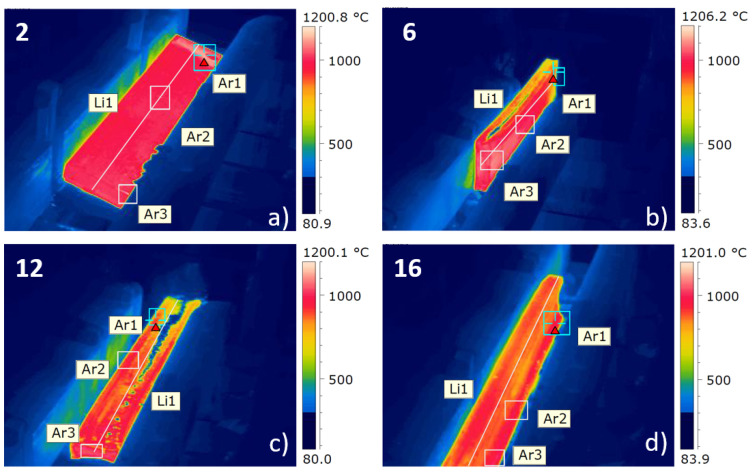
Representative thermographic images taken during the hot rolling process: (**a**) pass 2; (**b**) pass 6; (**c**) pass 12; (**d**) pass 16.The scale of the figures have been improved.

In this section, the results of simulations considering constant temperature of the beam for the whole process and varying temperature of the beam at each pass will be presented. A thermographic camera (FLIR^®^ systems, ThermaCAM^TM^ P65) was installed in the pulpit of the operator to obtain the real temperature of the beam during the hot rolling process. This information was fed into the numerical model in each pass to obtain results much closer to reality. Figure 6 shows some representative images of the thermographic data taken in the actual process.

Using the strategic points Ar1, Ar2 and Ar3 as well as the line Li1 (see Figure 6), the temperature was averaged to feed the numerical model to assign the corresponding temperature of the beam. It is important to mention that due to the position of the thermographic camera, it was only possible to obtain measurements of the odd passes. For the even passes, the average of the temperature of the preceding and following passes was used to complete the rolling sequence. Table 4 presents the temperatures used in each pass in the new lamination sequence. The results for these simulations were assigned the name “T.Real” in the following figures.

**Figure 7:**
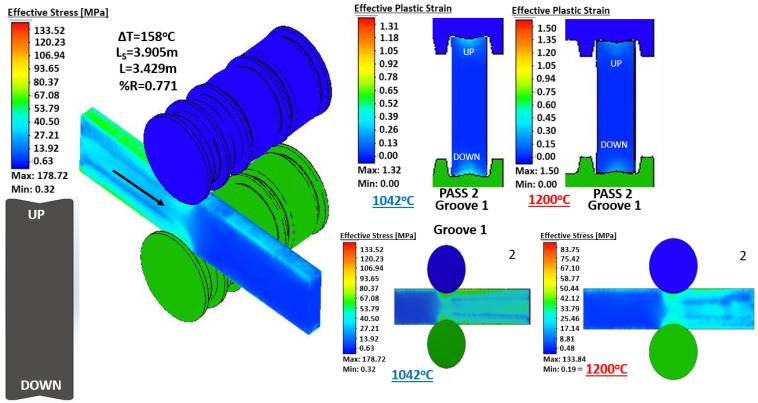
Comparison between hot rolling at constant temperature (1200 ${}^{\circ }C$) and real temperature for pass number 2.

Figure 7 shows the comparison of the simulation of hot rolling at a constant temperature (1200 ${}^{\circ }C$) and the real temperature obtained from measurements with the thermographic camera for pass number 2. Twenty-five similar images (one for each pass of the rolling sequence) were obtained to visualize and quantify the differences as a function of temperature, for the sake of space it is only shown a representative figure.

**Figure 8:**
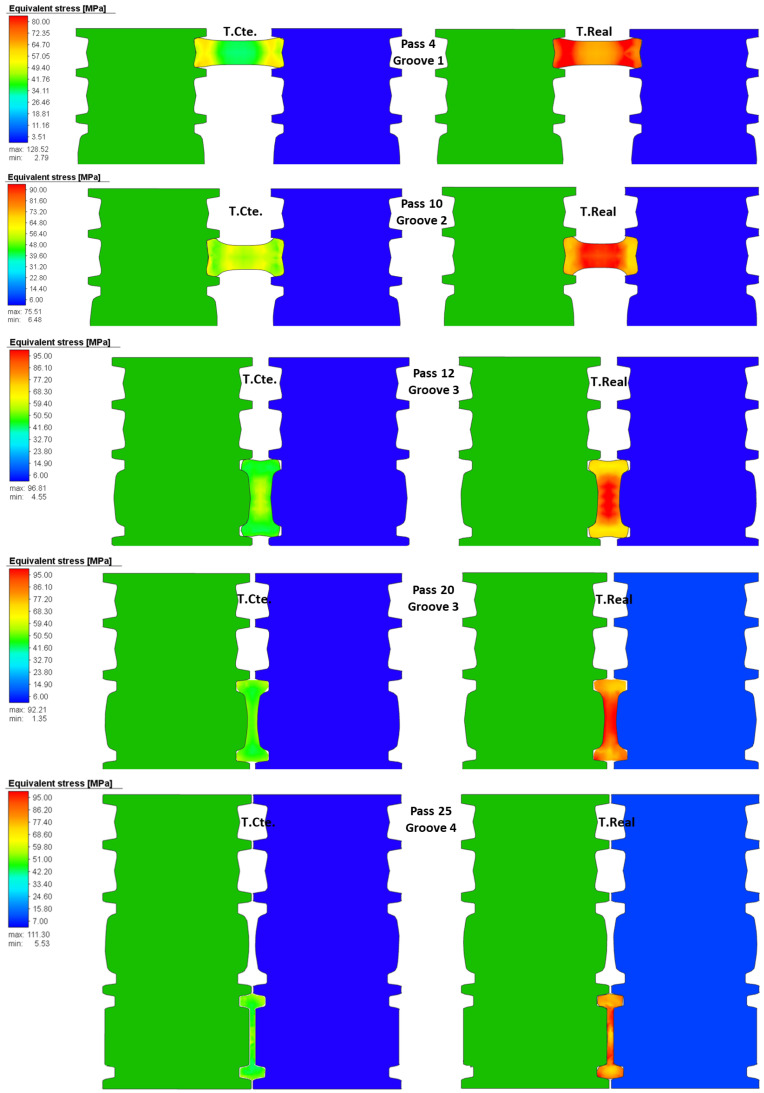
Distribution of effective stress in the middle of the beam during rolling of selected passes for simulations at constant and real temperature.

Figure 7 contains a lot of information, for example it is possible to observe the isometric of the virtual model representing the effective stress of the pass 2. It is possible to observe the thermal gradient between the ideal temperature and the real temperature (158 ${}^{\circ }C$), the final length predicted by the numerical model (3905 mm), the final length calculated analytically (3429 mm), and the percentage of reduction in area (0.771%). The upper right part of the image shows the comparison of the effective plastic strain for both cases (constant temperature and real temperature). Furthermore, finally, in the lower right part, the values of the effective stress are shown for both cases. An important feature of the numerical simulation is the posibility of obtaining information during the rolling process such as the effective stress in a cross-section. Figure 8 shows the distribution of effective stress in the central cross-section of the beam during the rolling of selected passes for the simulation at constant and real temperature. Note that measurements of this kind are imposible to obtain in-plant because it is not possible to section the beam during the rolling. The passes in Figure 8 were selected to show the rolling in the four grooves: pass 4 is performed in groove 1, pass 10 in groove 2, passes 12 and 20 in groove 3 (in this groove the desired I-profile is almost achieved), and pass 25 in groove 4.

**Figure 9:**
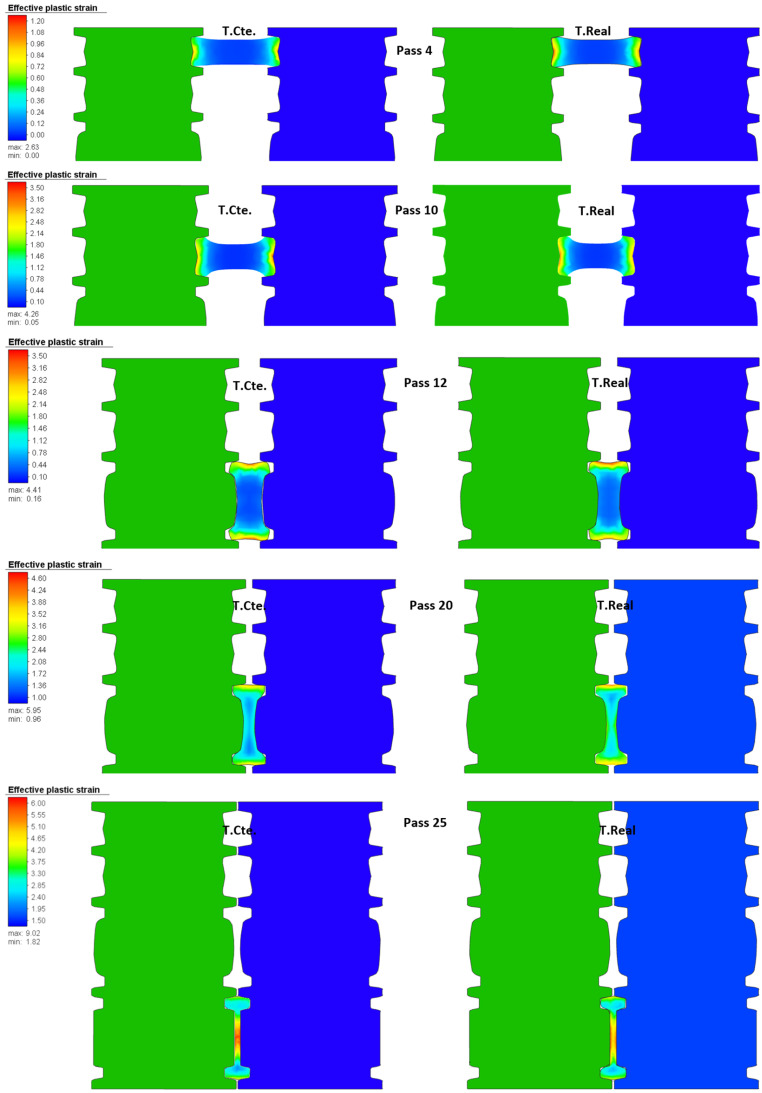
Distribution of effective strain in the middle of the beam during rolling of selected passes for simulations at constant and real temperature.

In Figure 8, higher values of effective stresses in the cross-section for the passes at real temperature can be observed. This is a consequence of rolling at a lower temperature (T.Real) since the material needs larger forces to deform and thus, resulting in higher stresses. A similar behavior was observed for the 25 passes. Figure 9 shows the distribution of effective strain in the central cross-section of the beam during the rolling at constant and real temperature for the same passes shown in Figure 8.

The passes for the simulations at constant and real temperature in Figure 9 show a similar distribution of the effective plastic strain. The similitude in the distribution can be attributed to the fact that the beam undergoes essentially the same deformation (i.e., the gap is the same regardless of the temperature of the simulation). A similar distribution is also observed for the rest of the passes.

**Figure 10:**
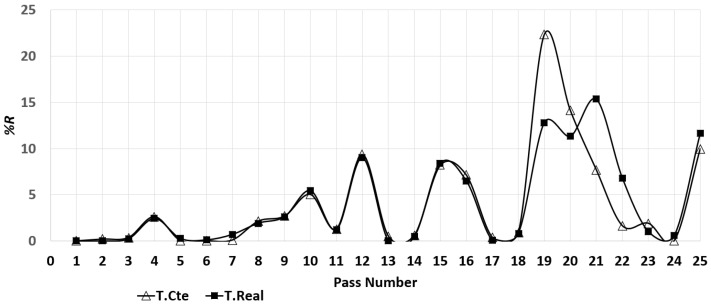
Evolution of the reduction of area (%R) for the simulations.

The reduction of area (%R) per pass was calculated for the simulations using Equation (9) and is shown in Figure 10. A good agreement can be observed for the simulations at constant and real temperature for the first 18 passes. The difference for passes 19–25 can be attributed to the temperature difference of the simulations, the beam cools as the rolling sequence progresses (see Table 4). Passes 1–4 are performed in groove 1 and the increment in %R is caused by the gradual reduction of gap (see Figure 3). At pass 5 the apparent drop in %R is due to the change of groove. By desing at passes 5 and 11 (change of groove) the gap has been adjusted to slightly deform the beam resulting in very small %R. Passes 5–10 are performed in groove 2 and %R increases as the gap is reduced to deform the beam (see Figure 3). For passes 11–18 the beam moves between grooves 2 and 3. The apparent peaks around passes 12 and 15 are generated because the beam moves back to groove 2 (in passes 13–14 and 17–18) with a $90^{\circ }$ rotation and the gap increased (see Figure 3) to deform only the sides of the beam, resulting in low %R. Passes 19–24 are done in groove 3 and a dominant peak in %R can be observed. At pass 19 the change of groove occurs with the beam rotated $90^{\circ }$ and the gap reduced. This change of orientation, gap and geometry is the cause of the significant increment of %R. The higher value for the simulation at constant temperature can be explained as a consequence of rolling at a higher temperature, since the material is easier to deform, filling a larger area of the groove than in the real temperature case. For passes 19–22 the gap is gradually reduced maintaining a relatively high %R, but for passes 22–24 the gap is the same (see Figure 3) resulting in low %R. Finally, in pass 25 there is a change in groove (from 3 to 4), this change of geometry generates an increment in %R.

The evolution of the power to accomplish the rolling of the beam is an important parameter, specifically the maximum power is monitored in-plant to avoid reaching the limits of the electric motors moving the rolls. Excesive power is not desirable during the process because it could damage the motors, mechanical parts or compromise the power supply of the facility. For the simulations, the power required to roll the steel can be calculated using Equation (11) [19].

(11) $$P=\frac{M\cdot n}{9550}$$

where *P* is the engine power, m is the engine torque and *n* is the revolutions per minute. The power in-plant is estimated using the voltage (*V*) and current (*A*) provided to the motors:

(12) P=V·A

**Figure 11:**
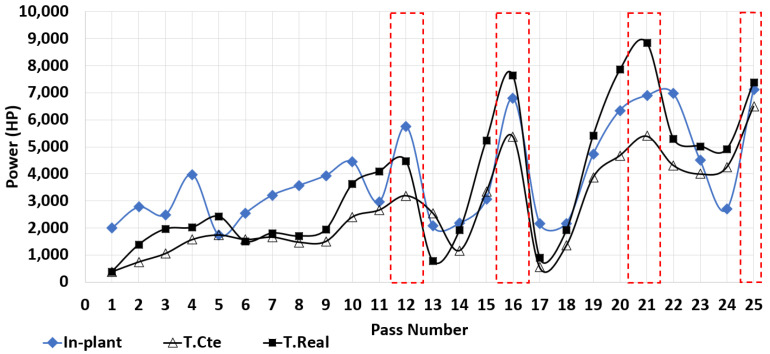
Maximum power for each pass of the rolling sequence for the simulations and in-plant measurements.

The maximum power for each pass for the simulations and in-plant measurements is shown in Figure 11. A good agreement can be observed between the simulations and in-plant measurements. The curves follow the same trend and exhibit 4 peaks (indicated in the graph with red dashed lines). The dominant peak occurs around pass 21 corresponding to groove 3. The highest values of maximum power were found in the simulation at real temperature. This overestimation of power is expected and results from the fact that the temperature of the entire beam at the beginning of each pass was assumed to be that of the surface in the real process (see Section 3). In the real process the beam is cooling (by convection and radiation) resulting in a distribution of temperatures with the center of the beam slightly hotter. Hence, the beam in the real process requires slightly lower power to deform the hotter material in the center compared to the simulation T.Real. The lowest values of power are for the simulation at constant temperature, this result is logical because at high temperature the material is easier to deform requiring lower power.

During passes 1–10 the beam undergoes slight deformations only to roughly form an I-shape, thus, requiring a relatively low power. The apparent peaks around passes 12 and 16 are formed because when the beam moves back to groove 2, the beam is rotated $90^{\circ }$ and the gap is increased to deform only the short sides of the beam (see Figure 8) requiring a low power. The passes of the dominant peak (19–24) in Figure 11 are performed in groove 3. The gap is gradually reduced in passes 19–22 requiring high power to roll the beam through groove 3 (with a more complicated geometry and larger area of contact compared with those of grooves 1 and 2). Then, the gap is maintained constant for passes 22–24 causing a sudden drop in power since the beam takes the I-shape of the groove. Finally, rolling with a different groove geometry and small gap creates the increase in required power in the last pass. Furthermore, note the similar form of the curves in Figure 10 and Figure 11, especially the location of the peaks. The resemblance can be explained as the fact that a large change in area of cross-section requires high power to roll the beam and attain the change in geometry.

**Figure 12:**
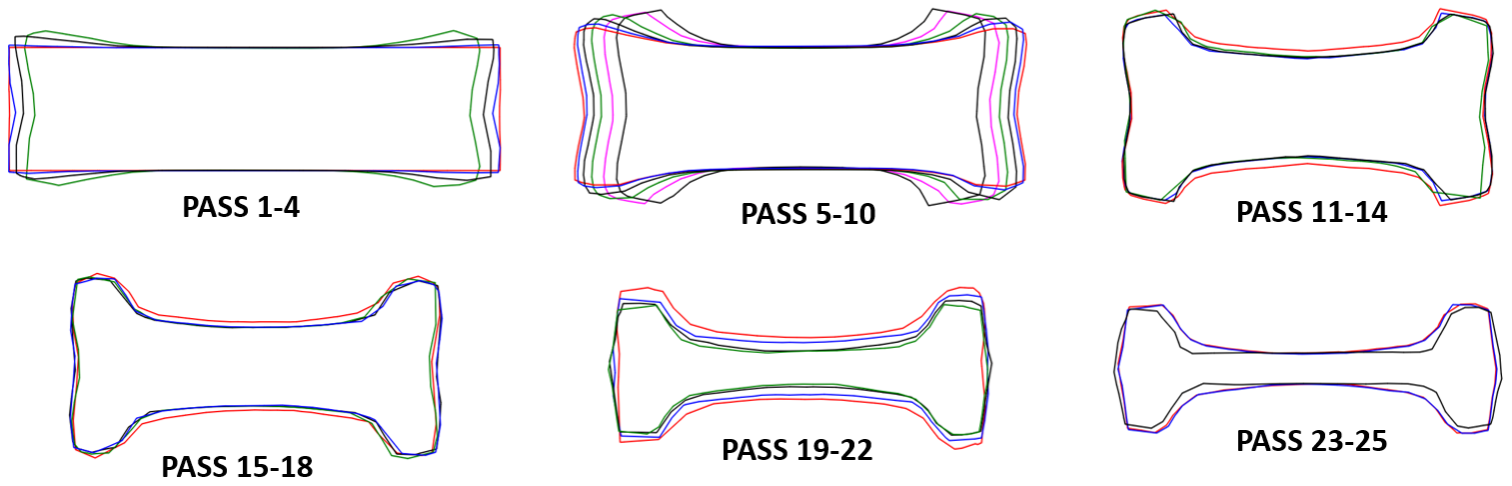
Geometry of the cross-sectional area at the center of the beam after each pass for the simulation at real temperature.

The simulations allow to examine the cross-sectional area of the beam after each pass to visualize the evolution of the geometry. Figure 12 shows the cross-section at the center of the beam for each pass for the simulation at real temperature. The cross-sections away from the center and those for the simulation at constant temperature are very similar to those of Figure 12, they are not shown for sake of clarity. Similar measurements in-plant would be expensive and time-consuming since the process needs to be ended at the corresponding pass and 25 beams would be waisted the company only allowed to section the beam of pass 25 and the dimensions will be compared below). The objective of the first 10 passes is to roughly form an I-shape deforming the short sides of the beam. The change of geometry of the short sides of the beam is evident in Figure 12 since this is the area of contact with the rolls (see Figure 8). Passes 11–12 are performed in groove 3 and a more defined I-shape of the cross-section can be observed because the rolls start to deform the central part of the beam (see Figure 8). Then, passes 13–14 are done in groove 2 with the beam rotated $90^{\circ }$ deforming only slightly the short sides of the beam. These observations are consistent with the formation of the first peak in power (Figure 11) and the graph of %R (Figure 10). Similarly, passes 15–16 are performed in groove 3 and then the beam moves back to groove 2 for passes 17–18. Observe the significant change in the area of cross section of passes 15–18 with respecto to passes 11–14. This change in geometry is consistent with the second peak in power (Figure 11) and the graph of %R (Figure 10) since high power is necessary to reduce the cross-section and results in large %R. Passes 19–24 are performed in groove 3 with the gap gradually reducing, note the significant reduction of cross-section compared with the geometry of passes 15–18. Such change of geometry requires high power to attain the deformation of the beam and is the reason of the formation of the dominant peak in required power (Figure 11) and the graph of %R (Figure 10). For passes 22–23 the gap is the same and the contours of the cross-section overlap resulting in low required power and %R. Finally, pass 25 is performed in groove 4 causing a reduction of cross-section resulting in the increase of required power and %R of Figure 11 and Figure 10, respectively.

**Figure 13:**
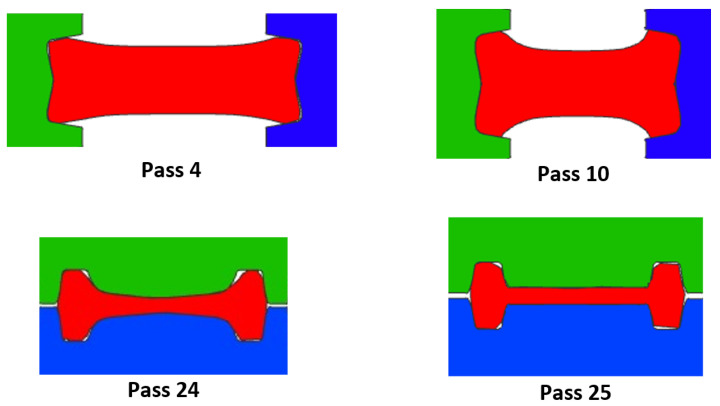
Central cross-section of the beam during the last pass at each groove for the simulation at real temperarure.

With the simulations it is possible to obtain cross-sections of the beam and the groove during the pass to observe the space filled by the material. Figure 13 shows cross-sections of the center of the beam and the grooves during the last pass for each groove to examine the maximum area covered by the beam. The beam closely acquires the shapes of grooves 1 and 2, indicating a very good design of groove’s geometry and rolling sequence. However, for groove 3 and 4, there are small unfilled areas for the corresponding last pass. Although the unfilled area is small, improvements might be possible modifying the geometry of groove 3 (e.g., chaging the radii of the contour) to generate a better flow of the material to fill the space. Groove 4 does not need modifications because it is used only in one pass and has already the desired geometry.

**Figure 14:**
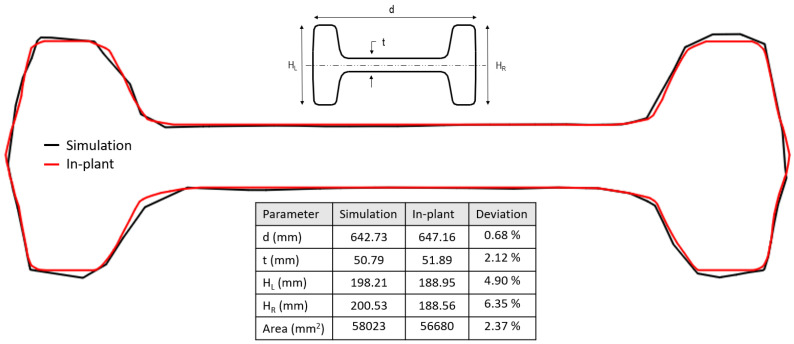
Comparison of the final cross-section of the beam for the simulation T.Real and in-plant measurements.

As mentioned above, only the beam of pass 25 was allowed to be sectioned to compare its geometry with the simulation’s result. Figure 14 shows the comparison of the central cross-section of the simulation and in-plant measurements. The contours of the simulations and in-plant measurements are in good agreement. The largest differences obtained are for $H_{L}$ (4.9%) and $H_{R}$ (6.35%), indicating a slight overestimation of the filling of the groove in the simulation in the vertical direction (see the orientation of the beam in Figure 13). The deviation in the area of cross-section is very small (2.37 %) and considering the excellent agreement in the shape of the cross-section, it can be concluded that the simulations are very accurate to predict the geometry of the beam in the actual process, especially considering the large number of passes simulated.

Lastly, although the rolling sequence is considered adequate to obtain an I-profile, based on the results some recommendations can be made to make the process more efficient. Especifically, if a reduction in the number of passes is possible, it would be beneficial for the process because it impacts directly in the time and cost of the production. The results show a minimal %R (Figure 10), very low power required to roll (Figure 11) and the geometry essentially unchanged compared with that of the previou pass (Figure 12) for passes 13, 17 and 24. The beam moves back to groove 2 in passes 13 and 17 to shape only the short sides, however, the opening of the gap seems excesive and passes 14 and 18 would be enough to complete this task. The gap is the same for passes 22–24 and when pass 24 is performed there is virtually no deformation on the beam. Hence, the recomendation of possibly eliminate passes 13, 17 and 24 of the rolling sequence can be made with confidence based on these findings. The reduction of passes implies a significant reduction of time and cost of the process considering the large number of beams manufactured, increasing the competitiviness of the company.

## 4. Conclusions

Based on the results of numerical simulations and measurements at the real process presented in this work, the following conclusions can be drawn:

1. The use of a FEM sofware to simulate 25 consecutive passes of hot rolling allowed to determine the distribution of effective stress, effective plastic strain, rolling moment, rolling power, final length and area of cross-section of the beam at each rolling pass. The results were in good agreement with those obtained from in-plant measurements.
2. The numerical simulation was validated comparing the geometry of the cross-section with in-plant measurements after the 25 passes, reporting a very similar shape and deviation of only 2.37% in area.
3. In the simulations, the reduction of cross-sectional area predicted unfilled areas between the work rolls, a common observation in the real process. This topic is very extensive and interesting to dedicate a future complete article in order to study the methodology of correct groove-roll design.
4. The incorrect metal heating before the rolling process might result in substantial quality defects and possible damage in the mill due to the excessive demand of power to roll the cold material. Proper deformation and metal flow depends, among others, on the uniform heating of the beam prior to the beginning of the rolling.
5. This study demonstrates the usefulness of the mathematical simulation to observe and quantify details essentially imposible to obtain in the production plant, which can be the key to obtain high quality products and minimize economic losses due to rejections and defects in production.
6. Using the present research methodology, it is possible to save time, money and have a better control of the process, generating the possibility of innovating in the production of profiles with more complex geometries and new materials.

## Figures and Tables

**Table 1:** Input parameters used for the three-dimensional FEM model.

Model Parameter	Value
Billet cross section	812 × 203 ${\mathrm{mm}}^{2}$
Billet length	3911 mm
Input billet temperature	1200 ${}^{\circ }C$
Initial temperature of rolls	65 ${}^{\circ }C$
Work roll diameter	1104.90 mm
Work roll barrel length	2184.40 mm
Heat transfer coefficient	50 $W/m^{2}K$
Coefficient of friction	0.3–0.4 in function of temperature
Thermal conductivity	26–29 W/m·K in function of temperature
Thermal expansion	$12\times 10^{-6}\;K^{-1}$
Billet us specific heat capacity	640–660 J/kg·K in function of temperature
Billet us density	7850 $\mathrm{kg}/m^{3}$
Poisson’s ratio of billet	0.3
Young’s modulus of billet	90–130 GPa in function of temperature
Revolutions per minute RPM	60–65 depending on pass

**Table 2:** Rolling schedule used in IPR profile manufacturing, gap in mm.

Pass	Gap	Hot Mill Direction	Groove	RPM	Beam Orientation
1	630	Forward	1	65	Initial orientation
2	612	Backward	1	65	
3	579	Forward	1	65	
4	541	Backward	1	65	
5	533	Forward	2	65	
6	523	Backward	2	65	
7	505	Forward	2	65	
8	477	Backward	2	65	
9	441	Forward	2	65	
10	398	Backward	2	65	$180^{\circ }$ clockwise rotation
11	149	Forward	3	60	$90^{\circ }$ clockwise rotation
12	124	Backward	3	60	
13	424	Forward	2	65	$90^{\circ }$ clockwise rotation
14	399	Backward	2	65	
15	105	Forward	3	65	$90^{\circ }$ clockwise rotation
16	80	Backward	3	65	
17	424	Forward	2	65	$90^{\circ }$ clockwise rotation
18	396	Backward	2	65	
19	58	Forward	3	65	$90^{\circ }$ clockwise rotation
20	33	Backward	3	65	
21	15	Forward	3	65	$180^{\circ }$ clockwise rotation
22	6	Backward	3	65	
23	6	Forward	3	65	$180^{\circ }$ clockwise rotation
24	6	Backward	3	65	
25	10	Forward	4	65	

**Table 3:** Analytical predictions of %R and final length per pass.

Pass	%R	L (mm)	Pass	%R	L (mm)
1	14.97	3402.26	14	8.39	5559.95
2	0.77	3428.68	15	8.93	5668.32
3	5.42	3625.43	16	12.9	6383.60
4	4.91	3812.91	17	13.1	6895.20
5	20.5	3164.17	18	12.45	7291.50
6	1.39	3208.84	19	12.45	7301.23
7	2.46	3290.00	20	16.92	8777.06
8	3.97	3426.25	21	14.26	10,237.22
9	5.27	3616.92	22	8.31	11,165.44
10	6.75	3878.94	23	0	11,165.44
11	14.38	4530.74	24	0	11,165.44
12	10.51	5063.07	25	11.62	12,653.51
13	10.51	5063.07			

**Table 4:** Average temperature used to feed the numerical model.

Pass Number	Temperature (${}^{\circ }$C)	Pass Number	Temperature (${}^{\circ }$C)
1	1049	14	904
2	1042	15	900
3	1035	16	916
4	1019	17	932
5	1003	18	917
6	948	19	902
7	892	20	882
8	934	21	863
9	975	22	869
10	947	23	875
11	919	24	872
12	913	25	869
13	907		

## Data Availability

Not applicable.

## References

[1] Kurt G., Yaşar N. (2020). Comparison of Experimental, Analytical and Simulation Results for Hot Rolling of S275JR quality Steel. J. Mater. Res. Technol..

[2] Kwon H.C., Im Y.T. (2002). Interactive Computer-Aided-Design System For Roll Pass and Profile Design in Bar Rolling. J. Mater. Process. Technol..

[3] Mori K., Osakada K., Oda T. (1982). Simulation of Plane-Strain Rolling by the Rigid-Plastic Finite Element Method. Int. J. Mech. Sci..

[4] Nalawade R.S., Marje V.R., Balachandran G., Balasubramanian V. (2016). Effect of Pass Shedule and Groove Design on the Metal Deformation of 38Mn VS6 in the Initial Passes of Hot Rolling. Sadhana.

[5] Ståhalberg U., Göransson A. (1986). Heavy Reductions by Means of ’Non-Bite’ Rolling, Including some Observations on Workpiece Shape. J. Mech. Work. Technol..

[6] Lee Y., Choi S., Kim H., Choo W.Y. (2000). An experimental study of the mean effective strain in rod (or bar) rolling process. Met. Mater. Int..

[7] Lee Y., Kim S.I., Choi S., Jang B.L., Choo W.Y. (2001). Mathematical model to simulate thermo-mechanical controlled processing in rod (or bar) rolling. Met. Mater. Int..

[8] Mróz S., Jagiela K., Dyja H. (2007). Determination of the energy and power parameters during groove-rolling. J. Achiev. Mater. Manuf. Eng..

[9] Shahani A., Nodamaie S., Salehinia I. (2009). Parametric Study of Hot Rolling Process by the Finite Element Method. Sci. Iran..

[10] Licheng Y., Jinchen J., Jinxiang H., Liwei N. (2011). Prediction of Process Parameters on Stress and Strain Field in Hot Rolling Process Using Finite Element Method. J. Inf. Technol..

[11] Nalawade R.S., Puranik A.J., Balachandran G., Mahadik K.N., Balasubramanian V. (2013). Simulation of Hot Rolling Deformation at Intermediate Passes and its Industrial Validity. Int. J. Mech. Sci..

[12] Braga F.V., Escobar E.P., Ávila T.J.R., de Oliveira N.J.L., Andrade M.S. (2016). Recrystallization of Niobium Stabilized Ferritic Stainless Steel During Hot Rolling Simulation by Torsion Tests. J. Mater. Res. Technol..

[13] Rout M., Pal S.K., Singh S.B. (2018). Prediction of edge profile of plate during hot cross rolling. J. Manuf. Process..

[14] Hanoglu U., Šarler B. (2018). Rolling Simulation System for Non-Symmetric Groove Types. Procedia Manuf..

[15] Rappaz M., Bellet M., Deville M.O., Snyder R. (2003). Numerical Modeling in Materials Science and Engineering.

[16] Duan X., Sheppard T. (2001). Prediction of Temperature Evolution by FEM During Multi-Pass Hot Flat Rolling of Aluminum Alloys. Model. Simul. Mater. Sci. Eng..

[17] Byon S.M., Kim S.I., Lee Y. (2004). Predictions of roll force under heavy-reduction hot rolling using a large-deformation constitutive model. Proc. Inst. Mech. Eng. Part B J. Eng. Manuf..

[18] Shah S.M., Nélias D., Coret M. (2012). Numerical simulation of grinding induced phase transformation and residual stresses in AISI-52100 steel. Finite Elem. Anal. Des..

[19] MSC Corporation (2019). Marc, Feature Pack 1, Volume A: Theory and User Information.

